# Systematic review of frameworks used to conceptualise health pathways of individuals diagnosed with cardiovascular diseases

**DOI:** 10.1136/bmjgh-2020-002464

**Published:** 2020-09-14

**Authors:** Maureen L Seguin, Avanti Rangnekar, Alicia Renedo, Benjamin Palafox, Martin McKee, Dina Balabanova

**Affiliations:** 1 Department of Health Services Research and Policy, London School of Hygiene and Tropical Medicine Faculty of Public Health and Policy, London, UK; 2 Social and Environmental Health Research, London School of Hygiene and Tropical Medicine Faculty of Public Health and Policy, London, UK; 3 Global Health and Development, London School of Hygiene and Tropical Medicine Faculty of Public Health and Policy, London, UK

**Keywords:** cardiovascular disease, health systems, public health

## Abstract

The treatment of cardiovascular disease (CVD) is managed inadequately globally. Theoretically informed frameworks have the potential to account for the multiple elements which constitute the CVD patient pathway, and capture their inter-relationships and processes of change. However, a review and critique of such frameworks is currently lacking. This systematic review aims to identify and critically assess frameworks of access to and utilisation of care which capture the pathways of patients diagnosed with one or more CVDs. The specific objectives are to (1) review how existing frameworks have been used and adapted to capture CVD patient pathways and (2) draw on elements of Strong Structuration Theory to critically appraise them, in terms of their ability to capture the dynamics of the patient journey and the factors that influence it. Five bibliographic databases were searched in January 2019. We included qualitative and quantitative studies containing frameworks used to capture the patient pathway of individuals with CVD, encompassing symptoms, diagnosis, treatment and long-term management. Data on patient behaviour and structural factors were interpreted according to elements of Strong Structuration Theory to assess frameworks on their ability to capture a holistic patient journey. The search yielded 15 articles. The majority were quantitative and all focused on management of CVDs, primarily hypertension. Commonly used frameworks included the common-sense self-regulation model, transtheoretical model and theory of planned behaviour. A critique drawing on elements of Strong Structuration Theory revealed these frameworks narrowly focused on patient attributes (patient beliefs/attitudes) and resulting patient action, but neglected external structures that interacted with these to produce particular outcomes, which results in an individualistic and linear view of the patient pathway. We suggest that a framework informed by Strong Structuration Theory is sufficiently flexible to examine the patient pathway, while avoiding a strict linear view facilitated by other frameworks.

**Key questions**

**What is already known?**

- Several theoretical frameworks have been used to understand the complex behaviours and care pathways of patients with cardiovascular disease (CVD).

**What are the new findings?**

- These existing frameworks are insufficient to fully grasp the nuances that characterise CVD patient behaviour and the factors which shape their actions over time.

**What do the new findings imply?**

- A framework drawing on elements from Strong Structuration Theory may provide the flexibility and comprehensiveness needed to overcome these deficits.

## Introduction

Cardiovascular disease (CVD) continues to be managed inadequately in countries at all levels of development.^1^ Many individuals diagnosed with CVD face barriers accessing affordable, high-quality, long-term care, and even if they overcome them, a high proportion soon fail to adhere to treatment. The reasons relate to the design of health systems, the health services that are provided, their responsiveness to patient needs and expectations and characteristics of the patients themselves. Many of these factors have been addressed in isolation, with limited success. As a consequence, attention is moving towards a holistic approach taking into account individual, institutional and structural factors and how these interrelate to shape patient pathways.^2^ This is the approach taken by the ‘Responsive and Equitable Health Systems—Partnership on Non-communicable Diseases’ (RESPOND) study, which aims to holistically explore pathways of hypertensive patients in Malaysia and the Philippines.^3^ Achieving this aim requires a critical analysis of existing frameworks which attempt to capture pathways of patients diagnosed with CVDs.

Patients with CVDs frequently come in and out of what are considered ideal or ‘standardised’ pathways. For instance, patients may discontinue medication, disengage with health services and/or develop alternative management to the ones planned by health services (eg, consume traditional medicine). Frameworks are beneficial insofar as they can illuminate a snapshot of the multiple elements (individual, sociocultural or health system-related) which constitute the pathway, and capture their inter-relationships and processes of change. Second, frameworks may be derived from theories which provide explanation of the observed phenomena. As such, they may represent an operationalisation of a set of ideas or theory. Frameworks also enable the exploration of patterns across multiple settings (within or across countries) and of underlying common patterns and obstacles along the pathway.

A range of theoretical frameworks have been developed to facilitate understanding of patient pathways,^4^ offering a broad picture of the multiple elements (individual or health system-related) which constitute the pathway. Theoretical frameworks have improved understanding of relationships between health beliefs and medication-taking behaviour.^5^ Treatment-adherent patients may be highly engaged with the health system, accessing professional help when needed, responding to professionals when contacted and closely following a set care plan. Meanwhile, poorly adhering patients may be less engaged or engage at different points than their adherent counterparts; although, in reality, the same patients may fall into both categories at different times, as circumstances within the health system, their personal life, family resources and logistic factors such as availability of transportation change.

A patient pathway is a construct that explains success or failure in patients’ progression through a planned health trajectory. When frameworks describing these pathways are too narrow (eg, focusing only on personal or biomedical aspects), they remove attention from broader sociostructural dimensions of health experiences and engagement with care,^6^ constructing patients as ‘the problem’. As such, frameworks imply presuppositions of who/what the problem is and produce particular representations of patients. A critical appraisal of existing frameworks focused on CVD patient pathways is necessary to illuminate such presuppositions and develop frameworks which facilitate a more holistic account of patient behaviour and social circumstances which may shape behaviour.

This paper presents the findings of a systematic review seeking to identify and critically assess frameworks of access to and utilisation of care which capture the pathways of patients diagnosed with one or more CVDs. The specific objectives are to (1) review how existing frameworks have been used and adapted to capture pathways followed by patients with CVD and (2) draw on elements of Strong Structuration Theory (SST) to critically appraise them, in terms of their ability to capture the dynamics of the patient journey and the factors that influence it. In the ‘Theoretical background’ section, we provide a description of the key elements of SST, starting by outlining Structuration Theory, its predecessor.

### Theoretical background

Sociological perspectives are useful for exploring the dynamic processes and mechanisms which mediate patient engagement with health systems, including barriers encountered.^7 8^ For instance, Structuration Theory, developed by Giddens in ‘The Constitution of Society’,^9^ focuses on micro-level, meso-level and macro-level factors to explain individual behaviour and features of society, and crucially, how these evolve over time.

Structuration Theory attempts to overcome two opposing approaches in the social sciences: the structuralist approach, which tends to overlook the potential of human action, and the individualistic approach, which tends to neglect the impact of social structures in shaping action.^9 10^ Giddens attempted to integrate these approaches. He defined structures as rules (procedures which perpetuate social life, including social conventions and official regulations) and resources (sources of power).^9^ Agency is human action, and involves individuals being knowledgeable about the rules or conventions that govern this action. Critically, Giddens proposed a ‘duality of structure’: that structures are created by, and shape, human agency.^11^ Thus, structures and agency are interdependent and mutually reinforcing, two sides of the same coin. This approach has enabled researchers to explore social phenomena at macrolevel mesolevel and microlevel.^12^

Structuration Theory does, however, have an important limitation in that empirical researchers have struggled to operationalise these concepts of structure and agency. Archer argued that structure and agency cannot be separated empirically in Giddens’s version of Structuration Theory, precluding the possibility of studying the independent properties of either.^13^ In a similar vein, Stones criticises Structuration Theory on the grounds that it treats agency and structure as abstract concepts only, limiting empirical application.^11^

**Figure 1:**
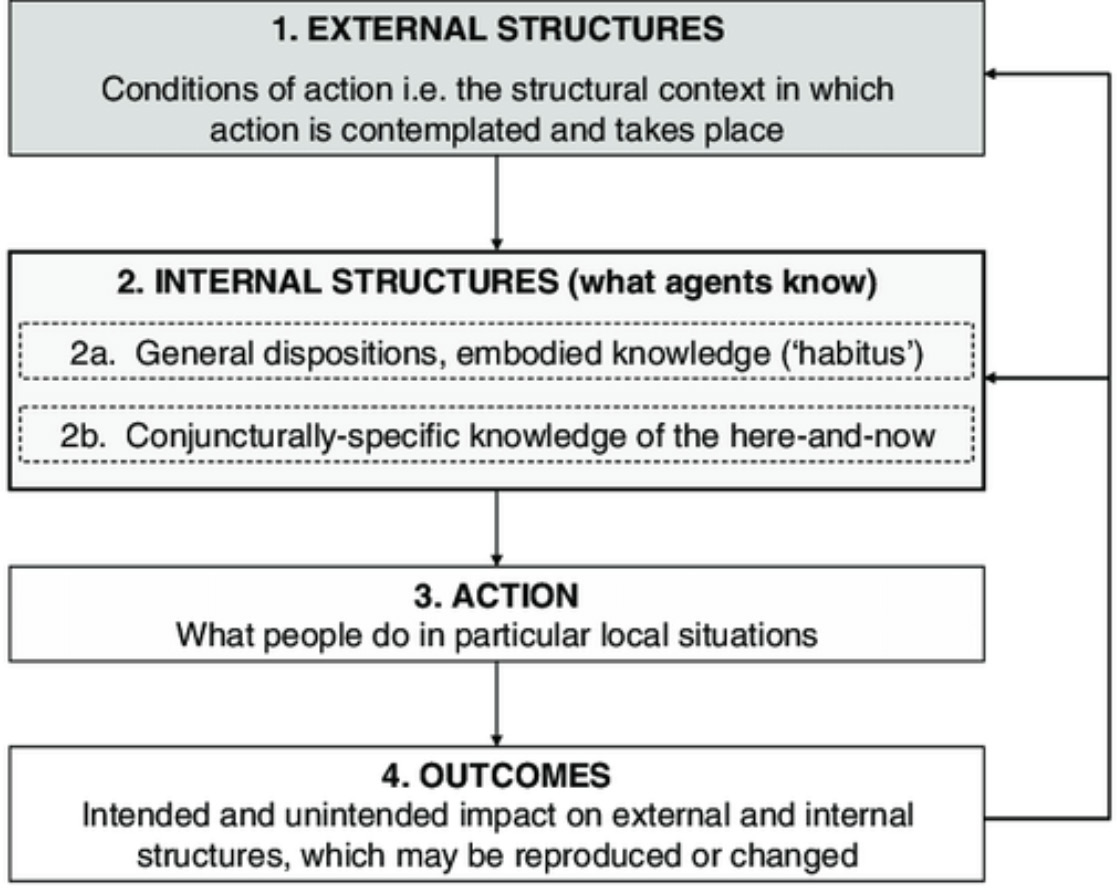
Elements of Strong Structuration Theory. Adapted from Stones.^11^

Progressing from these debates, Stones revised Structuration Theory and offered a tighter and clearer synopsis of the theory in order to guide empirical research, termed SST.^11^ The theory consists of four main elements (a ‘quadripartite cycle of structuration’) consisting of external structures, internal structures, action/active agency and outcomes.^11^ The elements are illustrated in figure 1.

External structures are autonomous to individuals, and include traditions, norms, moral codes and established ways of doing things. They include macro-level societal institutions such as health, legal and educational systems. Although these factors and institutions shape human behaviour (and human choice), this relationship is bi-directional; external structures are modified by the choices made by individuals.^11^ For instance, patients choosing to treat their hypertension with complimentary medicine perpetuates this industry. External social structures become internalised into individuals’ views of the social world, forming internal structures. Internal structures consist of two types: (i) *general dispositions* are the relatively enduring and transposable attitudes, opinions, beliefs and embodied knowledge of socially positioned individuals (similar to Bourdieu’s notion of habitus);^14^ this is the *generalised* knowledge of, and set of dispositions towards, the world, which individuals adapt to different situations; and these defining characteristics include the interpretative (or cultural/phenomenological) frames that inform their practices across conjunctures and situations; and (ii) *conjuncturally specific* internal structures comprises situationally *specific* knowledge concerning aspects of the immediate terrain of action; this kind of knowledge, which is always framed by the cultural frames of general dispositions, is often significantly altered by involvement in, and experience of, social practices within specific terrains, which themselves are typically in flux. The third element of SST is action/active agency, exercised by individuals drawing on their internal structures in order to act. Such actions produce outcomes, the fourth element of SST, either by preserving and reproducing structures faithfully, or changing them depending on the behaviour of the individual.^11 15^

The quadripartite cycle of structuration offers a vantage point from which to understand and interpret the non-linearity of patient pathways. CVD patients’ experiences with initial diagnosis and treatment, and their subsequent interaction with the health system could influence how the patient perceives ‘Western’ or alternative forms of treatment. Because SST proposes that internal structures are grounded in objective external structures, it provides the potential to better comprehend patient pathways and choices which are often misaligned with ideal or ‘standardised’ pathways. Due to this advantage presented by SST, we chose to draw on SST elements to critically appraise the articles included in this review.

## Methods

### Eligibility criteria

This review focuses on frameworks which have been developed and used to capture elements of the patient pathway (including symptoms, diagnosis, treatment commencement and long-term management) of individuals with one or more CVDs. No review protocol was developed prior to this work. The main inclusion criteria included papers which:

- featured frameworks with a longitudinal design (examined either prospectively or retrospectively), examining at least two junctures on the CVD patient pathway, from symptoms, diagnosis, treatment initiation and management (while this description of the pathway is linear, we recognise it is more complex);
- focused on management, but consider steps taken over time rather than at one point in time;
- featured unique frameworks empirically tested in studies of CVDs;
- significantly modified or updated an older framework;
- inductively proposed a new framework or combined elements from two or more frameworks arriving at significantly different theoretical models. This criterion, and the criterion bulleted above, yielded the full breadth of frameworks that had been used to illustrate CVD pathways, rather than an exhaustive compilation of papers using any framework. A set of articles containing such a compilation would have been too numerous to feasibly synthesise and was beyond the core aim of the paper.

The main exclusion criteria excluded papers which:

- drew on an existing framework without modifying it;
- presented frameworks but not fully describing their application in studies, or described framework components but neglected to describe interrelationships;
- described an intervention;
- presented frameworks which described service delivery or clinical pathways without capturing patient responses;
- described structural frameworks used in large-scale reform plans and service transformations;
- focused on acute or semi-acute CVDs, for instance, hypertensive conditions during pregnancy;
- were books, editorials, commentaries, poster presentations and protocol papers;
- were published in languages other than English.

### Search strategy

We followed an iterative process, progressively refining our focus and search strategy, drawing on the multidisciplinary nature of our team to incorporate a range of expertise to direct the search strategy.^16^ This organic process was appropriate for the exploratory nature of the review.

A preliminary search on Medline was conducted in June 2017, updated and broadened in a final database search conducted in January 2019, which also included PsychINFO, International Bibliography of the Social Sciences, Academic Search Complete and Web of Science databases. A full Medline search string is provided in the online supplementary material.

10.1136/bmjgh-2020-002464.supp1 Supplementary data

Filters were used to select English-language articles only, and to exclude articles published before 1980. Search terms covered aspects related to: CVDs (including heart failure, hypertension, heart disease and angina), quantitative and qualitative empirical studies, patient pathways and conceptual frameworks.

### Selection process

Selection of papers for inclusion followed the five stages in the preferred reporting items for systematic reviews and meta-analysis checklist.^17^ First, titles and abstracts identified in the initial Medline search as outlined above were downloaded into Endnote. Second, these were independently reviewed by two authors against the inclusion and exclusion criteria. Titles and abstracts identified in the final search in January 2019 were independently screened by two authors, and full texts screened by three authors. Full texts of articles were screened by MLS, AR and DB. Articles that fulfilled the inclusion criteria outlined above were included in the review. Disagreements were resolved by discussion within the research team. An in-depth review of included studies was conducted. As our review is focused on frameworks used in articles, rather than the robustness of studies themselves, quality of included articles was not assessed.

### Data extraction and analytic approach

Article aim, type of CVD, country of study (and classification of country by income status), setting, sample, methodology and elements of pathways captured (symptoms, diagnosis, treatment initiation and/or managements) were extracted from each article into an Excel spreadsheet. The specific framework(s) drawn on or created were identified and extracted, as well as details on how existing frameworks were modified. We also recorded whether the framework was used for deductive purposes (eg, to classify and interpret findings) or in an inductive way (eg, as emerging from the themes observed in findings).

The frameworks included in the articles were examined to assess the extent to which they captured aspects of patient behaviour and the structural factors impacting the patient pathway. The most commonly used frameworks were contextualised by tracing the origin of the framework, along with its key elements and critiques. At the final stage, data on patient behaviour and structural factors presented in the papers were interpreted and coded according to elements of Stones’ SST.^11^ Specifically, instances of agency, internal and external structures, and the impact of agency on structures and vice versa were extracted where reported.

### Patient and public involvement

Representatives for neither patients nor the public were involved in developing this paper. However, they were extensively involved in the wider study,^3^ which prompted this review.

## Results

### Study selection

**Figure 2:**
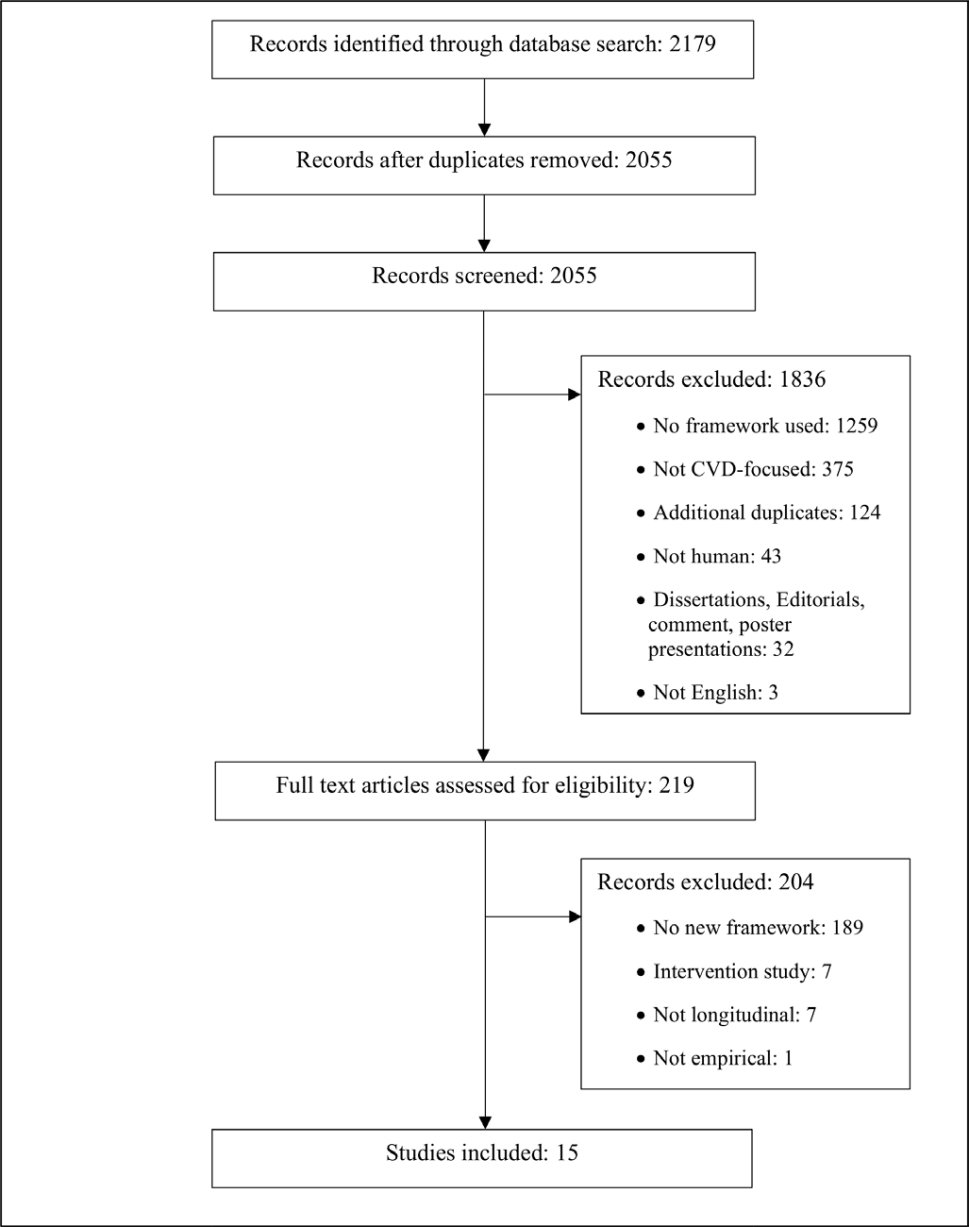
Results of the screening process. CVD, cardiovascular disease.

The results from the search are shown in figure 2. A total of 2179 records were identified at stage 1. After duplicates (n=124) were removed, titles and abstracts of 2055 were screened, yielding 219 full texts. This high number of full texts relative to the number of records initially identified reflects the challenge facing the research team as they sought to determine whether an innovative framework was used or developed in articles, which was frequently difficult to ascertain from abstracts and titles alone. A total of 204 were excluded due to lack of innovative framework, article type (notably interventions studies) and/or lack of empirical application of the framework. This left 15 for our review.

Details of the 15 included studies are reported in table 1. Although our scope was global, they were overwhelmingly from high-income countries. Nine were quantitative,^18–26^ four qualitative^27–30^ and two used mixed methods.^31 32^ Six focused on hypertension,^19–21 24 28 29^ three each on heart failure^26 27 31^ and coronary heart disease^18 23 25^ and there was one study each on congestive heart failure,^30^ acute coronary syndrome^22^ and myocardial infarction.^32^ Although we hoped to cover all parts of the CVD patient pathway, spanning symptoms, diagnosis, treatment initiation and long-term maintenance (including treatment adherence), the studies focused exclusively on long-term management.

**Table 1:** Characteristics of included articles

Study	Article aim	CVD	Country (income level)	Setting	Sample	Methodology	Framework details
Barello *et al*^27^	Identify features and the levers of patient with HF engagement	HF	Italy (HIC)	University Hospital in Milan	22 participants (13 patients, 5 physicians, 4 caregivers)	Qualitative, SSI Grounded theory.	Inductive, ‘Process of engagement in patients with HF’.
Bokhour *et al*^28^	Explore patients’ ‘explanatory models’ and context, relate to self-management	HT	USA (HIC)	Medical centres	48 African-American, white and Latino patients/veterans	Qualitative, SSI Grounded theory	Inductive, ‘The dynamic model of HT self-management behaviour’.
Byrne *et al*^18^	Describe illness perceptions and beliefs about medication of patients	CHD	Ireland (HIC)	General practice outpatients	1084 patients under the age of 80	Quantitative, cross-sectional, postal questionnaires	Deductive, SRM modified to include treatment and medication beliefs.
Chen *et al*^19^	Test relationship between illness perception to self-management	HT	Taiwan (HIC)	CVD clinics of teaching hospitals	355 patients	Quantitative, cross-sectional, structured questionnaires	Inductive/deductive: ‘Model for adherence to therapeutic regimens’, modified CSM.
Dickson *et al* 31	Examine contribution of attitudes, self-efficacy and cognition to management	HF	USA (HIC)	Outpatients of medical centre	41 patients	Mixed, qualitative SSI, quantitative cross-sectional survey	Deductive, modified Decision-making model of HF management.
Fort *et al* 29	Present patients’ perceptions of barriers and facilitators to management	HT and DB	Costa Rica/Mexico (UMIC)	Urban public health centres	70 patients	Qualitative, focus group discussions Thematic analysis	Deductive, TM.
Horowitz *et al*^30^	Elucidate patients' knowledge and beliefs, understand self-care routines	CHF	USA (HIC)	Urban tertiary care hospital	19 former inpatients	Qualitative, SSI, Grounded theory	Inductive/deductive: models of CHF and CSM.
Kressin *et al* 20	Explore links between race, beliefs about HT and adherence.	HT	USA (HIC)	Veteran's affairs hospital	793 outpatients (460 African-American, 333 white)	Quantitative, cross-sectional structured questionnaires	Deductive, adapted HDM combined with several other scales.
Luder *et al* 21	Describe features and beliefs of enrolees of employer-based DB and HT programme	HT and DB	USA (HIC)	Pharmacies	154 enrolees of employer-led DB and HT coaching programme	Quantitative, cross-sectional using survey	Deductive, HBM, TPB and TRA.
Peleg *et al* 22	Assess role of attitudes, norms and behavioural control on adherence	ACS	Israel (HIC)	Cardiac care units in urban hospitals	106 married/cohabitating male patients	Quantitative, longitudinal surveys	Deductive, TPB and Attachment Theory.
Platt *et al* 23	Examine adherence to medication, exercise and diet	CHD	Australia (HIC)	Outpatient clinics	142 outpatients	Quantitative, cross-sectional using questionnaire	Deductive, CSM, TM and positive and negative affect.
Presseau *et al* 32	Compare approaches for identifying determinants of adherence post-MI	MI	Canada (HIC)	Hospitals	24 outpatients for qualitative, 201 for quantitative	Mixed, qualitative SSI, quantitative: structured surveys	Deductive, TDF and HAPA.
Quine *et al* 24	Propose and test a model of adherence to antihypertensive medication	HT	UK (HIC)	Primary care	934 outpatients at 1 of 3 practices	Quantitative, prospective longitudinal using two surveys	Inductive, a conceptual model of adherence to HT medication.
Sniehotta *et al* 25	Test, compare, combine CSM and extended TPB	CHD	UK (HIC)	Hospitals, patient homes	103 outpatients in phase III cardiac rehabilitation	Quantitative, prospective cohort design	Deductive, CSM and TPB.
Vellone *et al* 26	Test situation-specific theory of HF self-care with modelling	HF	Italy (HIC)	Outpatient settings	417 outpatients aged 18 years and older	Quantitative, secondary analysis of data from cross-sectional study	Deductive, situation-specific theory of HF.

ACS, acute coronary syndrome; BP, blood pressure; CHD, coronary heart disease; CHF, congestive heart failure; CSM, common-sense self-regulation model; DB, diabetes; HAPA, health action process approach; HBM, health belief model; HDM, health decision model; HF, heart failure; HIC, high-income economy; HT, hypertension; LMIC, low-income to middle-income economy; MI, myocardial infarction; SRM, self-regulatory model; SSI, semi-structured interview; TDF, theoretical domains framework; TM, transtheoretical model; TPB, theory of planned behaviour; TRA, theory of reasoned action; UMIC, upper-income to middle-income economy.

### Frameworks to capture CVD patient pathways

Of the 15 included studies, 3 proposed a new framework, 10 drew on and modified existing frameworks and 2 used a combination of these approaches. The common-sense self-regulation model (and earlier self-regulation model) were the most commonly used, with five studies using them deductively.^18 19 23 25 30^ Three studies drew on the theory of planned behaviour^22 25^ or the earlier theory of reasoned action^21^ on which it is based. All of these studies incorporated elements of these theories with other existing frameworks.

Eight studies adopted a deductive approach and modified existing frameworks, including the health decision model,^20^ health belief model,^21^ theoretical domains framework^32^ or other models.^22 23 26 31 32^ Three studies proposed altogether new frameworks, following an inductive process.^24 27 28^ Additional details on these models appear in table 1.

As the common-sense self-regulation model, theory of planned behaviour and the transtheoretical model were the most commonly applied frameworks in our included articles, we next present an overview of key elements as originally developed in the three sections below entitled common-sense self-regulation model, theory of planned behaviour, and transtheoretical model. We show how each has two important limitations in conceptualising CVD patient pathways, so we seek an alternative. We show how Structuration Theory addresses some of the main limitations and discuss how it represents an advancement over the current types of framing.

#### Common-sense self-regulation model

The self-regulatory model, more recently known as the common-sense self-regulation model is a widely used theoretical framework for examining perceptual, behavioural and cognitive processes at play in self-management of health threats.^33 34^ It postulates that an individual’s perception of illness (termed cognitive representations) motivates the degree to which they take action to improve their health status.^35 36^ The original framework viewed individual perceptions of illness as resulting from five factors: illness identity (label or disease name and perceptions of associated symptoms), their causes (factors and/or behaviours predisposing an individual to the condition), consequences (personal impact of the condition, including experienced and anticipated physical, cognitive and social disruption), control (behaviours to regulate the condition) and timeline of the illness (individual perceptions about the duration of illness; chronological aspects of the disease course and management).^33 35–37^ More recent iterations of the framework separated the control factor into treatment control and personal control.

Proponents of the common-sense self-regulation model argue that the framework offers insights into individual differences in how illnesses are perceived, and how this influences behaviour regarding the illness.^23^ However, it has been criticised for the relative lack of attention to environmental and social factors in explaining patient behaviours.^38^ Moreover, it does not cover beliefs about medication or treatment, and how these may influence adherence.^39^ The authors acknowledge that the framework requires additional modification to fully grasp the dynamic nature of cognition and patient behaviour over time.^40^ For instance, Leventhal *et al*^33^ note:

The [common-sense self-regulation mode] is […] structured to describe transitions in behavior, that is, from nonadherence to adherence and from adherence to non-adherence, though few investigators have addressed the details of transitional processes. […] We need to identify and assess the variables and dynamic process involved in the initial transitions from discovering symptoms and labeling oneself as ill, to the intermediate term for seeking care and initiating treatment, to the transition from initiation to habitual performance of behaviors that is locked into daily behavioral patterns (pp. 936 and 944).

#### Theory of planned behaviour

The theory of planned behaviour, developed from the earlier theory of reasoned action^41^ seeks to predict and explain human behaviour in specific contexts.^42^ According to the former, health-related behaviour is a function of an individual’s intention to perform the action in question (termed behavioural intention).^42^ Intention results from three constructs: positive or negative attitude towards the action, perception of the social support or opposition to their fulfilling the action and individual perceptions of control over resources such as skills, confidence, and the ability to perform the behaviour. These elements influence an individual’s intentions, which then predict behaviour.

Critics have pointed out that the theory of planed behaviour is insufficient for predicting behaviour, as intentions can change during the period between the measurement of intention and the behaviour of interest.^43^ Thus, an intended behaviour may not be carried out. Some have suggested that forming action plans can bridge the intention-behaviour gap.^44^ Others have argued that factors beyond intention may influence behaviour, such as past behaviour, habits, self-identity and personality.^45 46^ A co-developer of the theory of planned behaviour emphasises that it should be modified if factors influencing intention beyond the original three constructs are identified.^42^

#### Transtheoretical model

The transtheoretical model of behavioural change, or the stages of change model, was proposed by Prochaska and DiClemente.^47^ Originally focusing on cessation of tobacco use, they suggested that behaviour change occurs in discrete stages: precontemplation (not thinking about stopping), contemplation (planning to stop between 31 days and 6 months in the future), preparation (planning to stop within 30 days), action (having stopped for 0–6 months) and maintenance (having stopped for >6 months).^47^ Another iteration included a sixth stage of change, termination (individual has permanently stopped).^48^ In all stages, individual self-efficacy is necessary to implement the behaviour change.^48^

Proponents of the transtheoretical model argue the model has enhanced health promotion interventions by highlighting the need to tailor interventions (beyond smoking cessation) according to the particular stage at which target individuals are situated, thereby improving effectiveness.^48–50^ Critics have argued that the time periods defining the stages are arbitrary,^51^ that the model unjustifiably assumes that individuals make coherent and stable plans^52^ and that important factors in behaviours change (such as reward and punishment) are unacknowledged.^53^ Nonetheless, authors have used constructs of the transtheoretical model to complement self-regulatory models (including the common-sense self-regulation model), arguing that this adds predictive value^23^ and enables a delineation between initial versus long-term behaviour change.^54^

#### Critique of current frameworks

The common-sense self-regulation model, transtheoretical model, theory of planned behaviour and theory of reasoned action share many common elements: all focus on the role of individual attitudes, perceptions and intention on subsequent individual behaviour,^4^ resulting in a representation of individual patient attributes as sole driving factors of pathways that depart from clinical guidelines. The impact of features of the health system (such as access, quality, patient-practitioner relations, health system department relations, etc) on medication adherence are beyond the explanatory scope of such frameworks. As a corollary, none of the frameworks above focus on how attitudes, intentions and perceptions are developed in the first place; they are treated as already-existing entities independent of the social world, including social, political, economic factors. It is increasingly recognised that individual behaviour is rooted in formal and informal structures, values and beliefs, and a focus solely on individuals obscures these influences.^55^

Efforts to integrate key elements from prominent theories of health behaviour have sought to address these issues, by inclusion of elements such as ‘environmental constraints’ (encompassing factors in the social environment) into explanatory models of health behaviour.^56^ However, even efforts to widen the group of variables (to include the social context) fail to address a fundamental issue: each framework takes an exclusively linear explanatory view of patient behaviour. Patient behaviour is taken as an end point to be explained, with independent factors including attitudes, preconceptions and intentions driving the behaviour of the patient as they progress towards that point. While patient behaviour and associated outcomes may be explained to some extent by these factors, patient behaviour is never static; patients may move between periods of adherence and non-adherence. The models do not articulate the ongoing consequences of these behaviours over time.

Given the limitations of existing frameworks, we have looked towards SST as it extends beyond individual-level explanations of patient behaviour (including the mesolevel and macrolevel), while recognising the non-linear nature of the pathways followed.

### Application of Strong Structuration Theory concepts to included articles

To explore the potential of SST to capture CVD patient pathways, we test the usefulness of the theory through applying its elements (external and internal structures, instances of patient agency and outcome) to the findings of the included articles (see online supplementary material, table 2). Articles are assessed according to the focus on agency, structure or both, and whether they propose a continuous (perpetual, never-ending) view on the patient pathway or a static view featuring an end-point cut-off. Seven articles focused primarily on structure,^18 20–25^ six on both agency and structure^19 27–30 32^ and two on agency exclusively.^26 31^ The frameworks in included articles overwhelmingly take a static approach to the patient pathway, with only one article adopting a continuous viewpoint.^30^

10.1136/bmjgh-2020-002464.supp2 Supplementary data

Articles considered the role of external structures including the healthcare system,^19 20 27 29 30^ social welfare system, work,^21 28^ physical environments,^28 29^ insurance companies,^21 30^ support system of family/friends,^30 32^ community educational resources on CVDs,^29^ social stigma against medication,^31^ societal patriarchal attitudes,^21 29^ social isolation^28^ and poverty.^18^ This wide array of structures indicates the healthcare system consists of (and is linked with) many kinds of external structures. Those structures beyond the healthcare system (eg, physical environments and patriarchal attitudes) are also potentially important factors in understanding patient behaviour and pathways.

Of the four SST elements, internal structures received the most attention, with all but two articles^19 26^ covering patient attitudes and beliefs. Eleven included patient attitudes toward treatment (including medication, diet and exercise regimes),^18 20–24 28–32^ with specific attention on attitudes towards food,^29^ prognosis^29 30^ and symptoms.^30^ Beliefs about the causes^28 30^ and nature of CVDs (specifically hypertension^18 20 28^ and coronary heart disease^25^) also featured prominently in the articles, along with attitudes towards health providers.^20 27 30 32^ Patient attitudes towards themselves were also explored, covering patient self-esteem,^27 29^ self-efficacy,^23 29 32^ confidence^21^ and self-identity.^24 32^ Finally, articles captured patient attitudes towards societal gender roles^29^ and stigma,^31^ as well as the role of religious beliefs^29^ on management. Despite the inclusion of these internal structures in articles, there was a relative lack of exploration of how they are influenced and formed by external structures.

In contrast, links between these internal structures and subsequent patient actions were explicit, with a wide variety of behaviours reported against the backdrop of these attitudes and beliefs. Most patient actions were framed as attempts to manage CVDs^18–20 23 26 27 29 31^ by making plans to control their condition^27 32^ on the basis of various forms of specific situational knowledge, such as monitoring symptoms,^26^ using medication reminders to develop a routine,^32^ addressing lifestyle risk factors,^23 28 29^ joining workplace heath programmes,^21^ seeking information on their condition and treatment^27^ and seeking advice and support.^29^ Some patients unilaterally chose to alter their hypertension medication on the basis of blood pressure readings,^28^ others declined medication^29–31^ or exercise regimes.^25^ One article reported that patients chose to present for care at emergency rooms rather than primary care.^30^

Of the four core concepts of SST, outcomes were focused on the least. Only one article touched on the impact of patient actions on structures, in this case external structures, by linking the tendency of patients to present at emergency rooms as perpetuating the treatment of congestive heart failure in hospitals rather than primary care.^30^

## Discussion

Conceptual frameworks can provide a tool that helps to make sense of the complex reality of CVD patient pathways, assisting in identifying the multiple interacting factors shaping it. This review draws on elements of SST to critically appraise existing CVD patient pathway frameworks, in terms of their ability to capture the dynamics of the patient journey and the factors that influence it. Despite the importance of models to understand relationships between health beliefs and medication-taking behaviour,^5^ we found relatively few papers that provided ones that capture the pathways followed by patients with CVD, spanning symptoms, diagnosis, treatment and long-term management. Moreover, the papers identified focused exclusively on the long-term management phase, completely excluding any consideration of earlier phases of the CVD pathway. The stage at which patients ‘enter’ the pathway is absent in the literature, although it is likely that a patient’s situationally specific experiences during initial symptoms, diagnosis and treatment initiation are likely to have an important and lasting impact on how they approach long-term maintenance.

An SST-informed framework has the advantage of allowing one to conceive of the patient pathway as a continuous process, considering how individual patient behaviour is shaped by their internalisation of external structures. Such an integrated vantage point is necessary to fully explicate the CVD patient pathway, which is typically marked by periods of adherence and non-adherence, characterised by shifting structures. Individuals increasingly access sources of information challenging Western medicine, including ideas coming from traditional medicine or unorthodox treatment for chronic conditions, making treatment choices in situations of increased complexity and decreased certainty.^57^ Factors influencing patient behaviours are not static.

A second advantage is the potential for SST to be used flexibly, including facilitating focus on earlier points in the patient pathway (eg, initial diagnosis). External structures relevant to the diagnosis phase may differ from later phases; SST facilitates exploration into transitions across these phases, allowing a view of patient involvement with healthcare systems as a sequential process rather than a one-off event.^58^ This is a major advance over the frameworks identified in this review, which overwhelmingly focus only on long-term management. With these advantages in mind, we suggest that future work on CVD patient pathways could benefit by drawing on SST elements.

An SST-informed framing allows for an exploration of pathways that starts with the patients and their engagement with structures and actors, demonstrating how a patient-centred approach can inform health systems development. Rather than suggesting a framework with a set number of elements and hierarchical relationships, we suggest a broad framing that can be useful for future work focused on CVD patient pathways which incorporates these elements and captures the health systems and broader structural issues affecting patient progression to a successful outcome. We propose an open-ended approach that builds on SST principles, offering a flexible lens through which to examine the intersecting factors affecting patient trajectory. Importantly, the proposed conceptual approach, with its flexibility and reconciliation of multiple influences on the patient journey, can help to arrive at context-specific and pragmatic policy solutions. These will posit the patient not just as a recipient of care but as a co-creator of health systems.^59^

The current heavy focus on the influence of individual patient beliefs and attitudes on action facilitated by current frameworks construct patients as rational (or non-rational) decision-makers, with little consideration of the factors which shape these beliefs and attitudes in the first place. However, in line with previous critiques of health psychology approaches typically used in public health,^60^ these external factors do constrain and enable patient behaviour, regardless of how rational or well-informed patients are. The current type of individual-level focus can result in strategies that focus exclusively on changing patients’ attitudes, knowledge and behaviour,^61^ rather than the environmental (social and health system-related) factors which may influence behaviour. To better reflect this lived patient reality, future intervention development to modify patient pathways to reflect clinical guidelines may benefit from patient involvement in co-designing pathways.^59^

This review has certain limitations. It focused only on academic articles published in English. The wide array of frameworks in the included articles made comparisons difficult. We reviewed 219 full texts, which is high relative to other systematic literature reviews. This is due to the nuanced nature of the topic which necessitated a manual approach to determine the presence or absence of appropriate frameworks.

## Conclusion

Existing frameworks used to understand pathways followed by patients with cardiovascular disease are insufficient to fully grasp the nuances that characterise patient behaviour and the factors which shape their actions over time. We propose that a framework drawing on elements from SST may overcome these deficits. The RESPOND study^3^ is doing this by applying SST concepts to examine hypertensive patient pathways in Malaysia and the Philippines.

## References

[1] Chow CK, Teo KK, Rangarajan S, et al Prevalence, awareness, treatment, and control of hypertension in rural and urban communities in high-, middle-, and low-income countries. JAMA 2013;310:959–68. 10.1001/jama.2013.184182 24002282

[2] Scott SE, Walter FM, Webster A, et al The model of pathways to treatment: conceptualization and integration with existing theory. Br J Health Psychol 2013;18:45–65. 10.1111/j.2044-8287.2012.02077.x 22536840

[3] Palafox B, Seguin ML, McKee M, et al Responsive and equitable health Systems-Partnership on non-communicable diseases (respond) study: a mixed-methods, longitudinal, observational study on treatment seeking for hypertension in Malaysia and the Philippines. BMJ Open 2018;8:e024000. 10.1136/bmjopen-2018-024000 PMC6067392 30061449

[4] Noar SM, Zimmerman RS Health behavior theory and cumulative knowledge regarding health behaviors: are we moving in the right direction? Health Educ Res 2005;20:275–90. 10.1093/her/cyg113 15632099

[5] Sabaté E Adherence to long-term therapies: evidence for action. Geneva, Switzerland: World Health Organization, 2003.

[6] Paparini S, Rhodes T The biopolitics of engagement and the HIV cascade of care: a synthesis of the literature on patient citizenship and antiretroviral therapy. Crit Public Health 2016;26:501–17. 10.1080/09581596.2016.1140127

[7] Frenz P, Vega J Universal health coverage with equity: What we know, don’t know and need to know, in Background paper for the global symposium on health systems research. First Global Symposium on Health Systems Research, Montreux, Switzerland, 2010.

[8] Goddard M Access to health care services--an English policy perspective. Health Econ Policy Law 2009;4:195–208. 10.1017/S174413310900485X 19187570

[9] Giddens A The constitution of society: outline of the theory of Structuration. Cambridge, UK: Polity Press, 1984.

[10] Rütten A, Gelius P The interplay of structure and agency in health promotion: integrating a concept of structural change and the policy dimension into a multi-level model and applying it to health promotion principles and practice. Soc Sci Med 2011;73:953–9. 10.1016/j.socscimed.2011.07.010 21849229

[11] Stones R Structuration theory. Basingstoke, UK: Palgrave Macmillan, 2005.

[12] Greenhalgh T, Stones R Theorising big IT programmes in healthcare: strong structuration theory meets actor-network theory. Soc Sci Med 2010;70:1285–94. 10.1016/j.socscimed.2009.12.034 20185218

[13] Archer MS Morphogenesis versus structuration: on combining structure and action. 1982. Br J Sociol 2010;61 Suppl 1): :455–83. 10.2307/589357 20092495

[14] Bourdieu P Outline of a theory of practice : Gellner E, Cambridge studies in social and cultural anthropology. Cambridge, UK: Cambridge University Press, 1977.

[15] Chan C, Deave T, Greenhalgh T Childhood obesity in transition zones: an analysis using structuration theory. Sociol Health Illn 2010;32:711–29. 10.1111/j.1467-9566.2010.01243.x 20545899

[16] Dixon-Woods M, Cavers D, Agarwal S, et al Conducting a critical interpretive synthesis of the literature on access to healthcare by vulnerable groups. BMC Med Res Methodol 2006;6:35. 10.1186/1471-2288-6-35 16872487 PMC1559637

[17] Moher D, Liberati A, Tetzlaff J, et al Preferred reporting items for systematic reviews and meta-analyses: the PRISMA statement. PLoS Med 2009;6:e1000097. 10.1371/journal.pmed.1000097 19621072 PMC2707599

[18] Byrne M, Walsh J, Murphy AW Secondary prevention of coronary heart disease: patient beliefs and health-related behaviour. J Psychosom Res 2005;58:403–15. 10.1016/j.jpsychores.2004.11.010 16026655

[19] Chen S-L, Tsai J-C, Chou K-R Illness perceptions and adherence to therapeutic regimens among patients with hypertension: a structural modeling approach. Int J Nurs Stud 2011;48:235–45. 10.1016/j.ijnurstu.2010.07.005 20678768

[20] Kressin NR, Wang F, Long J, et al Hypertensive patients' race, health beliefs, process of care, and medication adherence. J Gen Intern Med 2007;22:768–74. 10.1007/s11606-007-0165-9 17364243 PMC2219848

[21] Luder H, Frede S, Kirby J, et al Health beliefs describing patients enrolling in community pharmacy disease management programs. J Pharm Pract 2016;29:374–81. 10.1177/0897190014566311 25609662

[22] Peleg S, Vilchinsky N, Fisher WA, et al Personality makes a difference: Attachment orientation moderates Theory of Planned Behavior prediction of cardiac medication adherence. J Pers 2017;85:867–79. 10.1111/jopy.12294 27884040

[23] Platt I, Green HJ, Jayasinghe R, et al Understanding adherence in patients with coronary heart disease: Illness representations and readiness to engage in healthy behaviours. Aust Psychol 2014;49:127–37. 10.1111/ap.12038

[24] Quine L, Steadman L, Thompson S, et al Adherence to anti-hypertensive medication: proposing and testing a conceptual model. Br J Health Psychol 2012;17:202–19. 10.1111/j.2044-8287.2011.02034.x 22107150

[25] Sniehotta FF, Gorski C, Araújo-Soares V Adoption of community-based cardiac rehabilitation programs and physical activity following phase III cardiac rehabilitation in Scotland: a prospective and predictive study. Psychol Health 2010;25:839–54. 10.1080/08870440902915915 20204953

[26] Vellone E, Riegel B, D'Agostino F, et al Structural equation model testing the situation-specific theory of heart failure self-care. J Adv Nurs 2013;69:2481–92. 10.1111/jan.12126 23521633

[27] Barello S, Graffigna G, Vegni E, et al 'Engage me in taking care of my heart': a grounded theory study on patient-cardiologist relationship in the hospital management of heart failure. BMJ Open 2015;5:e005582. 10.1136/bmjopen-2014-005582 PMC4369000 25776041

[28] Bokhour BG, Cohn ES, Cortés DE, et al The role of patients' explanatory models and daily-lived experience in hypertension self-management. J Gen Intern Med 2012;27:1626–34. 10.1007/s11606-012-2141-2 22821569 PMC3509311

[29] Fort MP, Alvarado-Molina N, Peña L, et al Barriers and facilitating factors for disease self-management: a qualitative analysis of perceptions of patients receiving care for type 2 diabetes and/or hypertension in San José, Costa Rica and Tuxtla Gutiérrez, Mexico. BMC Fam Pract 2013;14:131–9. 10.1186/1471-2296-14-131 24007205 PMC3846574

[30] Horowitz CR, Rein SB, Leventhal H A story of maladies, misconceptions and mishaps: effective management of heart failure. Soc Sci Med 2004;58:631–43. 10.1016/S0277-9536(03)00232-6 14652059 PMC4301306

[31] Dickson VV, Deatrick JA, Riegel B A typology of heart failure self-care management in non-elders. Eur J Cardiovasc Nurs 2008;7:171–81. 10.1016/j.ejcnurse.2007.11.005 18178132

[32] Presseau J, Schwalm JD, Grimshaw JM, et al Identifying determinants of medication adherence following myocardial infarction using the theoretical domains framework and the health action process approach. Psychol Health 2017;32:1176–94. 10.1080/08870446.2016.1260724 27997220

[33] Leventhal H, Phillips LA, Burns E The Common-Sense model of self-regulation (CSM): a dynamic framework for understanding illness self-management. J Behav Med 2016;39:935–46. 10.1007/s10865-016-9782-2 27515801

[34] Hagger MS, Orbell S A meta-analytic review of the Common-Sense model of illness representations. Psychol Health 2003;18:141–84. 10.1080/088704403100081321

[35] Leventhal H, Meyer D, Nerenz D The common sense representation of illness danger : Rachman S, Contributions to medical psychology. New York: Pergamon Press, 1980: 7–30.

[36] Leventhal H, Nerenz D The assessment of illness cognition : Karoly P, Measurement strategies in health. New York: John Wiley & Sons, 1985: 517–54.

[37] Meyer D, Leventhal H, Gutmann M Common-sense models of illness: the example of hypertension. Health Psychol 1985;4:115–35. 10.1037/0278-6133.4.2.115 4018002

[38] Li W-W, Stotts NA, Froelicher ES Compliance with antihypertensive medication in Chinese immigrants: cultural specific issues and theoretical application. Res Theory Nurs Pract 2007;21:236–54. 10.1891/088971807782427967 18236769

[39] Horne R Representations of medication and treatment: advances in theory and measurement : Petrie K, Weinman J, Perceptions of health and illness: current research and applications. Amsterdam: Harwood Academic, 1997: 155–87.

[40] Leventhal H et al Living with chronic illness: a contextualized, self-regulation approach : Sutton S, Baum A, Johnston M, et al, The Sage handbook of health psychology. London, UK: Sage Publications, 2004: 197–240.

[41] Fishbein M, Ajzen I Belief, attitude, intention, and behavior: an introduction to theory and research. Reading, MA: Addison-Wesley, 1975.

[42] Ajzen I The theory of planned behavior. Organ Behav Hum Decis Process 1991;50:179–211. 10.1016/0749-5978(91)90020-T

[43] Sutton S Predicting and explaining intentions and behavior: how well are we doing? J Appl Soc Psychol 1998;28:1317–38. 10.1111/j.1559-1816.1998.tb01679.x

[44] Sniehotta FF Towards a theory of intentional behaviour change: plans, planning, and self-regulation. Br J Health Psychol 2009;14:261–73. 10.1348/135910708X389042 19102817

[45] Conner M, Armitage CJ Extending the theory of planned behavior: A review and avenues for further research. J Appl Soc Psychol 1998;28:1429–64. 10.1111/j.1559-1816.1998.tb01685.x

[46] Harris J, Hagger MS Do basic psychological needs moderate relationships within the theory of planned behavior? J Appl Biobehav Res 2007;12:43–64. 10.1111/j.1751-9861.2007.00013.x

[47] Prochaska JO, DiClemente CC Stages and processes of self-change of smoking: toward an integrative model of change. J Consult Clin Psychol 1983;51:390–5. 10.1037/0022-006X.51.3.390 6863699

[48] Prochaska JO, Velicer WF The transtheoretical model of health behavior change. Am J Health Promot 1997;12:38–48. 10.4278/0890-1171-12.1.38 10170434

[49] Singer EA The transtheoretical model and primary care: "The Times They Are A Changin'". J Am Acad Nurse Pract 2007;19:11–14. 10.1111/j.1745-7599.2006.00189.x 17214862

[50] Burkholder G, Evers K, Burbank P Application of the transtheoretical model to several problem behaviors : Burbank P, Rieb D, Promoting exercise and behavior change in older adults: interventions with the Transtheoretical model. New York: Springer, 2002: 85–145.

[51] Sutton S Back to the drawing board? A review of applications of the transtheoretical model to substance use. Addiction 2001;96:175–86. 10.1046/j.1360-0443.2001.96117513.x 11177528

[52] West R Time for a change: putting the Transtheoretical (stages of change) model to rest. Addiction 2005;100:1036–9. 10.1111/j.1360-0443.2005.01139.x 16042624

[53] Baumeister R, Heatherton T, Tice D Losing control: how and why people fail at self-regulation. San Diego: Academic Press, 1994.

[54] Maes S, Karoly P Self-regulation assessment and intervention in physical health and illness: A review. Appl Psychol 2005;54:267–99. 10.1111/j.1464-0597.2005.00210.x

[55] Hanefeld J, Powell-Jackson T, Balabanova D Understanding and measuring quality of care: dealing with complexity. Bull World Health Organ 2017;95:368–74. 10.2471/BLT.16.179309 28479638 PMC5418826

[56] Fishbein M The role of theory in HIV prevention. AIDS Care 2000;12:273–8. 10.1080/09540120050042918 10928203

[57] Cockerham WC, Rütten A, Abel T Conceptualizing contemporary health lifestyles: Moving beyond Weber. Sociol Q 1997;38:321–42. 10.1111/j.1533-8525.1997.tb00480.x

[58] Haenssgen MJ, Ariana P Healthcare access: a sequence-sensitive approach. SSM Popul Health 2017;3:37–47. 10.1016/j.ssmph.2016.11.008 29349202 PMC5769022

[59] Donetto S, Pierri P, Tsianakas V, et al Experience-based Co-design and healthcare improvement: Realizing participatory design in the public sector. Design J 2015;18:227–48. 10.2752/175630615X14212498964312

[60] Hepworth J The emergence of critical health psychology: can it contribute to promoting public health? J Health Psychol 2006;11:331–41. 10.1177/1359105306063298 16774882

[61] Zuiderent-Jerak T Situated intervention: sociological experiments in health care : Bijker W, Pinch T, Slayton R, Inside technology. London, England: The MIT Press, 2015.

