# Supplementary material for: Systematic review of frameworks used to conceptualise health pathways of individuals diagnosed with cardiovascular diseases
Source: BMJ Glob Health. 2020 Sep 14;5(9):e002464. doi: 10.1136/bmjgh-2020-002464 (PMC7490945; doi:10.1136/bmjgh-2020-002464)
Supplement: Supplementary data [file bmjgh-2020-002464supp001.pdf]

Online Supplemental material: MEDLINE search completed January 2019

1. exp Hypertension/ (242834)

2. high blood pressure.mp. (12278)

3. hypertens*.ti,ab. (362504)

4. (essential adj3 hypertension).ti,ab. (22542)

5. (isolat* adj3 hypertension).ti,ab. (1923)

6. (elevat* adj3 blood adj pressur*).ti,ab. (9419)

7. (high adj3 blood adj pressur*).ti,ab. (13652)

8. (increase* adj3 blood pressur*).ti,ab. (20957)

9. ((systolic or diastolic or arterial) adj3 pressur*).ti,ab. (165843)

10. or/1-9 (491108)

11. cardiac failure.mp. [mp=title, abstract, original title, name of substance word, subject heading word, keyword heading word, protocol supplementary concept word, rare disease supplementary concept word, unique identifier, synonyms] (10749)

12. chf.mp. [mp=title, abstract, original title, name of substance word, subject heading word, keyword heading word, protocol supplementary concept word, rare disease supplementary concept word, unique identifier, synonyms] (11799)

13. congestive heart failure.mp. [mp=title, abstract, original title, name of substance word, subject heading word, keyword heading word, protocol supplementary concept word, rare disease supplementary concept word, unique identifier, synonyms] (35932)

14. coronary heart disease.mp. [mp=title, abstract, original title, name of substance word, subject heading word, keyword heading word, protocol supplementary concept word, rare disease supplementary concept word, unique identifier, synonyms] (43878)

15. ischemic heart disease.mp. [mp=title, abstract, original title, name of substance word, subject heading word, keyword heading word, protocol supplementary concept word, rare disease supplementary concept word, unique identifier, synonyms] (22447)

16. heart failure/ (108069)

17. coronary disease/ (129612)

18. myocardial ischemia/ (37051)

19. or/11-18 (315992)

20. exp Angina Pectoris/ (42493)

21. exp Angina, stable/ (1081)

22. exp Angina, Unstable/ (10734)

23. or/20-22 (42493)

24. exp cardiovascular diseases (2241191)

25. 10 or 19 or 23 or 24 (2443330)

26. Epidemiologic studies/ (7842)

27. exp case control studies/ (963922)

28. exp cohort studies/ (1813364)

29. Case control.tw. (98854)

30. (cohort adj (study or studies)).tw. (139534)

31. Cohort analy$.tw. (5595)

32. (Follow up adj (study or studies)).tw. (42612)

33. (observational adj (study or studies)).tw. (71608)

34. Longitudinal.tw. (180607)

35. Retrospective.tw. (384033)

36. Cross sectional.tw. (242838)

37. Cross-sectional studies/ (283226)

38. qualitative research/ or exp interviews as topic/ or exp questionnaires/ or health care surveys/ or (qualitative or interview* or focus group* or theme* or questionnaire* or survey*).ti,ab. (1657266)

39. or/26-38 (3632723)

40. *"continuity of patient care"/ or *patient navigation/ (9967)

41. continuity of care.mp. (5279)

42. medication adherence.mp. or Medication Adherence/ (18489)

43. referral.mp. or "Referral and Consultation"/ (120483)

44. treatment pathway*.mp. (797)

45. clinical pathway*.mp. (2624)

46. critical pathways/ (6137)

47. care pathway*.mp. (2774)

48. disease management.mp. or Disease Management/ (40375)

49. patient compliance.mp. or Patient Compliance/ (60170)

50. regular provider*.mp. (119)

51. regular treatment*.mp. (1015)

52. care trajector*.mp. (178)

53. transition* of care.mp. (2375)

54. 40 or 41 or 42 or 43 or 44 or 45 or 46 or 47 or 48 or 49 or 50 or 51 or 52 or 53 (256718)

55. (Framewok or frame*).mp. [mp=title, abstract, original title, name of substance word, subject heading word, keyword heading word, protocol supplementary concept word, rare disease supplementary concept word, unique identifier, synonyms] (300673)

56. Concept Formation/ or concept.mp. (248910)

57. model.mp. (1569039)

58. dimension.mp. (55600)

59. typology.mp. (4055)

60. directed acyclic graphs, dag.mp. (8)

61. conceptual model.mp. (5240)

62. 55 or 56 or 57 or 58 or 59 or 60 or 61 (2076840)

63. 25 and 39 and 54 and 62 (1782)

64. limit 63 to (english language and humans and yr="2017-Current") (245)
