# Supplementary material for: Systematic review of frameworks used to conceptualise health pathways of individuals diagnosed with cardiovascular diseases
Source: BMJ Glob Health. 2020 Sep 14;5(9):e002464. doi: 10.1136/bmjgh-2020-002464 (PMC7490945; doi:10.1136/bmjgh-2020-002464)
Supplement: Supplementary data [file bmjgh-2020-002464supp002.pdf]

Online Supplementary material: Table 2. Application of Strong Structuration Theory concepts to included papers

Article	Framework emphasis*/ chronicity**	External structures	Internal structures	Agency	Outcomes
Barello et al. (2015)	B/S	Healthcare system, primarily GPs and cardiologists.	Attitudes on health providers: view doctor as a 'trusted ally' available to them when needed.<br><br>Attitudes toward themselves: autonomous and skilled actor in their care. A 'new normality' outlook enters patient's life, a state of well-being achieved through previous development and growth.	Patients make life plans, seek information on their condition and treatment, and are able to manage their condition.	N/A
Bokhour et al. (2012)	B/S	Social welfare system, social isolation, work schedules, unsafe physical environment in which to exercise.	Beliefs on causality of HT: genetics, stress, poor diet (excessive salt), lack of exercise. Spikes in BP caused by stress, pain, and exercising.<br><br>Beliefs on nature of HT: intermittent (not chronic) condition, not serious compared to DB or HIV.<br><br>Attitudes on treatment: should be modified according to symptoms, reluctance to take medication long-term due to side effects, perception of medication as unnatural and harmful, preference to treat through relaxation.	Some patients addressed lifestyle stressors believed to cause hypertension (adjusting foods or ignoring dietary and exercise advice), altering medication according to latest BP reading or symptoms (decreasing dosage after low readings, increasing after perceived symptoms), neglecting to increase medication on advice from medical professionals, engaging in relaxation or exercise, modifying exercise in response to the unsafe physical environment.	N/A
Byrne, Walsh & Murphy (2005)	S/S	Poverty	Beliefs on nature of HT: Chronic versus intermittent problem.<br><br>Attitudes on treatment: Medications are necessary/unnecessary, are harmful/not harmful.	Patients who believed HT was chronic were more adherent to those with other beliefs on the nature of HT.<br><br>Patients who believed medications were necessary and unharmful were more adherent.	N/A
Chen, Tsai & Chou (2011)	B/S	Health care systems characterised by a variety of options, including Western, traditional Chinese and folk medicine.	N/A	Some patients adhered, especially those who experienced symptoms, and felt they could control HT.	N/A
Dickson, Deatrick & Riegel (2008)	A/S	Stigma associated with taking medication.	Attitudes on treatment: positive attitude toward and confidence on management. View that that symptoms, causes, and treatment were linked. Attitudes toward water pills differed, with some having favourable views and some negative.<br><br>Perception of stigma: Patients believed that others perceived that only fat/lazy people got heart problems.	Those with a negative view of water pills were less adherent to them.<br><br>Those who were bothered by perceived stigmatisation of heart problems were less adherent to medication.<br><br>Patients developed habits to manage their condition, requiring less thought.	N/A

Fort et al. (2013)	B/S	Health care system characterised by poor communication between professionals and patients on the chronic nature of HT. Some instances of good care. Lack of medicines and follow-up was noted, and of accessibility due to price of services and medication.<br><br>Physical environment in which patients lived, characterised by a lack of space in which to exercise.<br><br>Community resources such as educational sessions for HT.<br><br>Patriarchal views in society; female role to care for other family members.	Attitudes on treatment: medication did not have to be taken long-term.<br><br>Attitude on prognosis: a fatalistic attitude was held by some participants, which acted as a barrier to adherence.<br><br>Attitude on food: a negative perception of healthy food as being expensive or unsatisfying.<br><br>Attitudes towards themselves: a desire to take care of oneself, loving oneself. High self-esteem and an attitude of self-efficacy encouraged adherence.<br><br>Attitudes toward gender roles: women should care for other family members, rather than adhere to their own treatment plans.<br><br>Belief in organised religion: Religious faith/placing faith in God to cure encouraged adherence.	Some patients stopped their medication to see how their bodies reacted.<br><br>Patients sought advice and support from other patients.<br><br>Some patients refuse to believe in their diagnosis, and hope it will spontaneously resolve.<br><br>Some patients accept their diagnosis, which encouraged adherence.<br><br>Patients may choose to diet and adhere to medications due to high self-esteem.<br><br>Patients choosing to stay calm and limiting stress exposure encouraged adherence.	N/A
Horowitz, Rein & Leventhal (2004)	B/C	Health care system characterised by inadequate information shared by professionals to patients on the causes, symptoms, management, and consequences of CHF.<br><br>Barriers created by insurance companies for patients attempting to access care outside emergency rooms (relative lack of primary care provision). Lack of accessibility due to lack of insurance cover for certain medications.<br><br>Family members or friends recognised symptoms, assessed severity of illness and need for help, interpreted medication regimens and ensured diets were followed, contacted doctors and took note of explanations and instructions.	Attitudes on health providers: Assumption that doctor should not be challenged by patients.<br><br>Attitude on prognosis: CHF viewed as a short-term, unserious, curable condition. Lack of understanding of the timeline, causes and consequences of CHF.<br><br>Attitudes on treatment: Most thought medications were effective and reported adherence. Lack of understanding of what medications were for, and that they had to be taken even in the absence of obvious symptoms. Most believed that the emergency room was the most effective place to receive treatment, because tests, technology and treatment were immediately available.<br><br>Attitudes on symptoms: Patients typically did not attribute symptoms to CHF. Worsening symptoms unrecognised or perceived as vague sensations of multiple possible, but unknown cause, and while bothersome, of unclear or low importance. Few participants believed that they could control symptoms, which was perceived as their physicians' responsibility.<br><br>Beliefs on causality of CHF: caused by stress or asthma, unrelated to heart function.	Widespread lack of management of symptoms by patients, until treatment at emergency room required/sought. Insurance issues led some patients to choose to deteriorate to qualify for a hospital admission to avoid the hassle of applying for coverage in primary care.<br><br>Patients did not have the tools to notice or address their worsening condition. Very few weighed themselves regularly (which helps monitor fluid retention). Participants who did recognize worsening symptoms often could not determine how to obtain care outside of an emergency room.<br><br>Most patients could not explain the cause of symptoms, due to lack of knowledge.	Lack of action on behalf of the patient to act on worsening symptoms led patients to present at emergency rooms, rather than management in primary care. Perpetuates treatment in emergency rather than in primary care.

Kressin et al. (2007)	S/S	Health care system characterised by inequality in counselling and advice provided by professionals to patients, with African Americans receiving more advice on adherence and link between HT and other CVDs than White counterparts. African Americans were more likely to receive written materials and were prescribed more types of medication. Doctors of White patients were more likely to request a follow-up appointment.	Attitudes on health providers: White patients had a higher degree of trust in their physicians than African American counterparts.<br><br>Attitude on treatment: African Americans were more likely to believe that medications would make them feel better, and to rate high BP as a serious matter.<br><br>Beliefs on nature of HT: African Americans were more knowledgeable about their high BP and treatment than their White counterparts.	No significant differences observed regarding adherence between African American and White patients.	N/A
Luder et al. (2016)	S/S	Employer-led DB and HT coaching program, characterised by financial incentives for those employees who join. Lack of awareness of the program was a barrier to joining.<br><br>Male employees were more likely to be referred to the program than female.	Attitudes towards themselves: Patients confident in performing health behaviours were more likely than unconfident patients to control their condition(s); this was especially true for African Americans. Single patients were less confident to make lifestyle changes than married.<br><br>Attitude on treatment: African American patients were more likely than White patients to believe the program help them manage their condition. African American patients placed greater importance on maintaining a healthy lifestyle than White patients.	Many patients chose to enrol due to newsletter adverts and financial incentives (especially White patients).<br><br>Those over the age of 65 were more likely to respond to newsletters from their insurance company advertising the program than younger colleagues.	N/A
Peleg et al. (2017)	S/S	N/A	Attitude on treatment: Intention to adhere and positive attitude toward treatment was associated with adherence amongst patients with lower attachment anxiety (those who worried that their partner might be unavailable or unsupportive).	N/A	N/A
Platt et al. (2014)	S/S	N/A	Attitude toward themselves: Self-efficacy was significantly correlated with mediation, diet, and exercise adherence.<br><br>Attitude on treatment: Higher diet self-efficacy was significantly independently associated with higher dietary adherence.	Patients were most adherent to medication, followed by dietary advice and exercise recommendations.	N/A
Presseau et al. (2017)	B/S	Social support from family and/or friends predicted medication adherence.	Attitudes on health providers: high level of trust in professionals facilitated adherence.<br><br>Attitude toward themselves: Self-efficacy predicted intention to take medication. The prospect of taking medication indefinitely challenged patients' self-identity, creating a barrier to adherence.<br><br>Attitude toward treatment: Belief that taking medication was beneficial predicted adherence.	Some patients engaged in Action Planning, which predicted adherence.<br><br>Some patients used reminders, which predicted adherence.<br><br>Adopting a routine including medication-taking was linked to adherence.	N/A

Quine et al. (2012)	S/S	N/A	Attitudes toward themselves: conscientiousness, having a hypertensive identity and perceived behavioural control predicted adherence.<br><br>Attitudes on treatment: intention to adhere and previous adherence predicted adherence.	N/A	N/A
Sniehotta, Gorski & Araujo-Soares (2010)	S/S	N/A	Beliefs on nature of CHD: The more participants believed their heart condition was cyclical rather than chronic, the less physical activity they engaged in.	Attrition in a cardio rehab was high, with more than 60% of participants not attending the final phase in a program.	N/A
Vellone et al. (2013)	A/S	N/A	N/A	Some patients monitored their symptoms, which predicted symptom recognition treatment adherence and evaluation.	N/A

* A = Agency, S = Structure, B = Both

** C = Continuous, S = Static
